# Transfection of poly(I:C) can induce reactive oxygen species-triggered apoptosis and interferon-β-mediated growth arrest in human renal cell carcinoma cells via innate adjuvant receptors and the 2-5A system

**DOI:** 10.1186/1476-4598-13-217

**Published:** 2014-09-17

**Authors:** Nanae Harashima, Takafumi Minami, Hirotsugu Uemura, Mamoru Harada

**Affiliations:** Department of Immunology, Shimane University Faculty of Medicine, 89-1 Enya-cho, Izumo, Shimane, 693-8501 Japan; Department of Urology, Kinki University School of Medicine, 377-2 Ohno-higashi, Osaka-Sayama, Osaka, 589-8511 Japan

**Keywords:** Poly(I:C), Reactive oxygen species, Renal cell carcinoma, Caspase-2, 2-5A system

## Abstract

**Background:**

Synthetic double-stranded RNA poly(I:C) is a useful immune adjuvant and exhibits direct antitumor effects against several types of cancers. In this study, we elucidated the mechanisms underlying the effects induced in poly(I:C)-transfected human renal cell carcinoma (RCC) cells.

**Results:**

In contrast to the lack of an effect of adding poly(I:C), poly(I:C) transfection drastically decreased RCC cell viability. Poly(I:C) transfection induced reactive oxygen species (ROS)-dependent apoptosis in RCC cells and decreased the mitochondrial membrane potential (ΔΨm). Treatment with *N*-acetyl-l-cysteine (NAC), a ROS scavenger, suppressed apoptosis and restored the ΔΨm. Although the levels of phosphorylated γH2A.X, an indicator of DNA damage, increased in poly(I:C)-transfected RCC cells, NAC treatment decreased their levels, suggesting ROS-mediated DNA damage. Furthermore, poly(I:C) transfection increased the levels of phosphorylated p53, NOXA, and tBid. Immunoblots and assays with a panel of caspase inhibitors revealed that poly(I:C) transfection-induced apoptosis was dependent on caspase-8 and -9, as well as caspase-2. Alternatively, poly(I:C) transfection increased mRNA expression of interferon (IFN)-β, and treatment with IFN-β suppressed growth of RCC cells without apoptosis. In addition, cyclinD1 and c-Myc expression decreased in poly(I:C)-transfected RCC cells. Moreover, RNA interference experiments revealed that poly(I:C) transfection exerted apoptotic effects on RCC cells through innate adjuvant receptors and the 2-5A system, the latter of which induces apoptosis in virus-infected cells.

**Conclusions:**

These results suggest that poly(I:C) transfection induced two types of effects against RCC cells such as apoptosis, as a result of ROS-mediated DNA damage, and IFN-β-mediated growth arrest, both of which were exerted via innate adjuvant receptors and the 2-5A system.

**Electronic supplementary material:**

The online version of this article (doi:10.1186/1476-4598-13-217) contains supplementary material, which is available to authorized users.

## Background

Immune adjuvant receptors, including Toll-like receptors (TLRs), play a crucial role in initiating and activating innate and adaptive immune responses [1]. Therefore, several TLR ligands have been utilized in anticancer vaccines as immune adjuvants with the expectation of augmenting antitumor immune responses in patients with cancer [2]. Intriguingly, some types of cancer cells express TLRs, and TLR ligands provide pro-survival or pro-apoptotic signals to cancer cells [3]. Lipopolysaccharide, a TLR4 ligand, provides pro-survival signals to cancer cells, thereby inducing therapy resistance [4, 5], whereas a synthetic double-stranded RNA (dsRNA) poly(I:C), a ligand for membrane-bound TLR3, shows direct antitumor effects on a variety of cancers [6–8]. Additionally, transfecting poly(I:C) into human melanoma cells induces apoptosis via melanoma differentiation-associated gene (MDA)5, a cytoplasmic poly(I:C) receptor [9, 10]. We have also reported that poly(I:C) transfection exhibits apoptotic effects against human breast cancer cells, whereas recovery of their viability by selective MDA5 knockdown is only partial [11]. The mechanism by which apoptosis is induced and the identities of receptors and/or mechanisms other than MDA5 that participate in the effects in poly(I:C)-transfected cancer cells have not been fully elucidated.

Apoptosis is triggered mainly through the extrinsic or intrinsic caspase-dependent pathways, and caspase-8 and -9, respectively, play central roles in these pathways [12]. Activation of caspase-8 transforms Bid to tBid, thereby promoting mitochondrial-mediated caspase-9-dependent apoptosis [13]. When cancer cells are exposed to anticancer drugs or radiation, changes in pro-apoptosis or anti-apoptotic molecules occur on the mitochondrial membrane, leading to activation of caspase-9 [12]. Alternatively, reactive oxygen species (ROS) induce intrinsic apoptosis by triggering DNA damage [14]. Notably, caspase-2 has been suggested to participate in ROS-mediated cellular apoptosis [15], but the precise roles of this “orphan” caspase in cancer cell apoptosis have not been fully explained [16].

Cellular apoptosis after viral infection is an antiviral mechanism that eliminates infected cells and prevents virus from spreading. Several receptors and mechanisms are involved in initiating cell death in response to viral infection. The 2-5A system is one of them and is composed of the 2′,5′ oligoadenylate synthetase (2-5OAS) and 2-5A-dependent RNase (RNaseL), both of which are enzymes that play crucial roles in antiviral defense mechanisms [17]. 2-5OAS is activated by dsRNA, an intermediate agent in the virus replication life cycle, to produce 2-5A (2′,5′ oligoA), thereby activating RNaseL [18]. Activation of the 2-5A system induces degradation of ribosomal RNAs and apoptosis in mammalian cells [19]. However, whether the 2-5A system is involved in the antitumor effects induced in poly(I:C)-transfected human cancer cells has not been determined.

In the present study, we elucidated the underlying mechanisms of the drastic effects of poly(I:C) transfection on human renal cell carcinoma (RCC) cells. Our results suggest that poly(I:C) transfection induces two types of effects against RCC cells: apoptosis, as a result of ROS-mediated DNA damage, and interferon (IFN)-β-mediated suppression of cell growth. Additionally, besides caspase-8 and -9, caspase-2 played important roles in the effects. Furthermore, the effects induced in poly(I:C)-transfected RCC cells were mediated not only by innate adjuvant receptors but also by the 2-5A system.

## Results

### Apoptosis in RCC cells after poly(I:C) transfection

**Figure 1:**
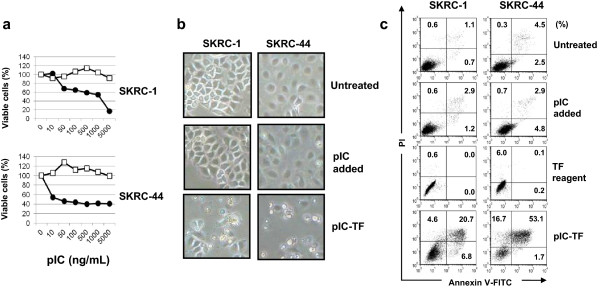
**Poly(I:C) transfection can induce apoptosis in RCC cells. (a)** RCC cells were cultured with poly(I:C) (white squares) or transfected with poly(I:C) (black circles) at the indicated doses. Cell viability was determined after 24 h using the WST-8 assay. Similar results were obtained from three independent experiments. **(b)** RCC cells were cultured or transfected with poly(I:C) at a dose of 1000 ng/ml for SKRC-1 cells and 500 ng/ml for SKRC-44 cells. Morphological changes were observed microscopically after 24 h. Magnification, ×40. Similar results were obtained from two independent experiments. **(c)** RCC cells were cultured or transfected with poly(I:C) (500 ng/ml for SKRC-1 and 50 ng/ml for SKRC-44). These cells were stained after 24 h with FITC-conjugated Annexin V and PI, and then analyzed by flow cytometry. Numbers represent the percentages of each subset. Similar results were obtained from five independent experiments. pIC, poly(I:C); pIC-TF, poly(I:C) transfection.

We first examined the effects induced when poly(I:C) was added or transfected into the two RCC lines (Figure 1a). Although adding poly(I:C) showed no definite effect on cell viability, poly(I:C) transfection drastically decreased viability in a dose-dependent manner. SKRC-44 cells were more susceptible to poly(I:C) transfection than that of SKRC-1 cells. Figure 1b shows the morphological changes. Although adding poly(I:C) induced no change in these cell lines, poly(I:C) transfection induced cell death, as observed by the non-adherent and shrunken cells. Flow cytometry after Annexin V/propidium iodide (PI) staining revealed that poly(I:C) transfection clearly increased the percentages of Annexin V^+^ cells in both SKRC-1 and SKRC-44 cells compared with those in the other groups (Figure 1c).

### ROS generation and decreased mitochondrial membrane potential (ΔΨm) in poly(I:C)-transfected RCC cells

**Figure 2:**
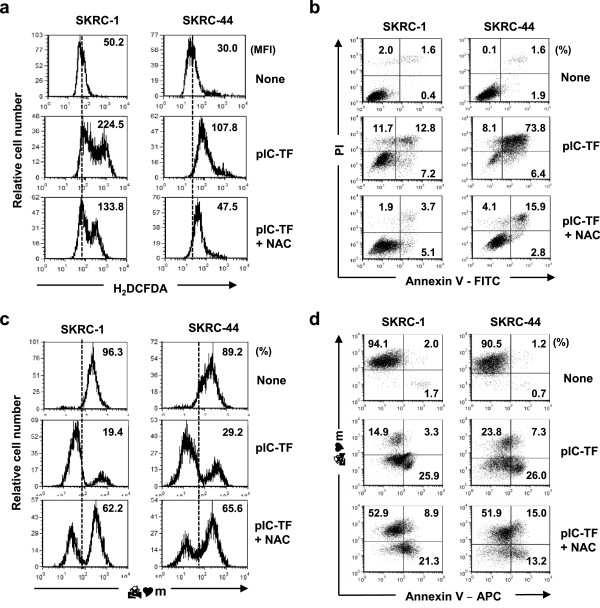
**ROS generation and decreased ΔΨm in poly(I:C)-transfected RCC cells. (a)** RCC cells were transfected with poly(I:C) (SKRC-1, 500 ng/ml; SKRC-44, 50 ng/ml) and cultured for 24 h in the presence or absence of NAC (10 mM). Treated cells were stained with carboxy-H_2_DCFDA. Numbers represent the mean fluorescence intensity (MFI) of cells in each subset. Similar results were obtained from three independent experiments. **(b)** Cells were then stained with Annexin V-FITC and PI and analyzed by flow cytometry. Numbers represent the percentages of cells in each subset. **(c)** RCC cells were transfected with poly(I:C) (SKRC-1, 1,000 ng/ml; SKRC-44, 500 ng/ml) and cultured for 24 h in the presence or absence of NAC (10 mM). Similar results were obtained from three independent experiments. RCC cells were then examined for ΔΨm levels by flow cytometry using Alexa Fluor 488-conjugated MitoProbe™ DiOC_2_(3) (8 nM). Numbers represent the percentages of positive cells with ΔΨm^high^. Similar results were obtained from three independent experiments. **(d)** RCC cells were then stained with Alexa Fluor 488-conjugated DiOC_2_(3) (8 nM) and Annexin V-APC and analyzed by flow cytometry. Numbers represent the percentage of positive cells in each subset. Similar results were obtained from two independent experiments. pIC-TF, poly(I:C) transfection.

As ROS are known to be involved in intrinsic apoptosis [20], we next determined whether poly(I:C) transfection triggered the generation of ROS, whose levels were evaluated using 5-(and-6)-carboxy-2′, 7′-dichlorohydrofluorescein diacetate (carboxy-H_2_DCFDA), a specific ROS-detecting fluorescent dye. As a result, staining intensity of carboxy-H_2_DCFDA increased in both cell lines after poly(I:C) transfection, and this increase was inhibited by adding the ROS scavenger *N*-acetyl-l-cysteine (NAC) (Figure 2a). The NAC treatment apparently decreased the percentages of Annexin V^+^ cells in poly(I:C)-transfected SKRC-1 and SKRC-44 cells (Figure 2b), indicating that ROS comprise a key mediator of apoptosis after poly(I:C) transfection.

We next examined the level of ΔΨm in these cells because its level is reversely associated with the intrinsic apoptotic pathway [21]. We examined ΔΨm levels using the cyanine dye DiOC_2_(3) and found that the percentages of cells with a high ΔΨm decreased by poly(I:C) transfection; these decreased percentages were partially restored by NAC treatment (Figure 2c). We further investigated the relationship between ΔΨm level and Annexin V-staining. As shown in Figure 2d, poly(I:C) transfection increased Annexin V^+^ RCC cells with a low ΔΨm, and the NAC treatment conversely decreased such cells but increased Annexin V^-^ RCC cells with a high ΔΨm. These results indicate that poly(I:C) transfection induced ROS generation and thereby induced apoptosis in RCC cells in association with decreased ΔΨm.

### ROS-mediated DNA damage in poly(I:C)-transfected RCC cells

**Figure 3:**
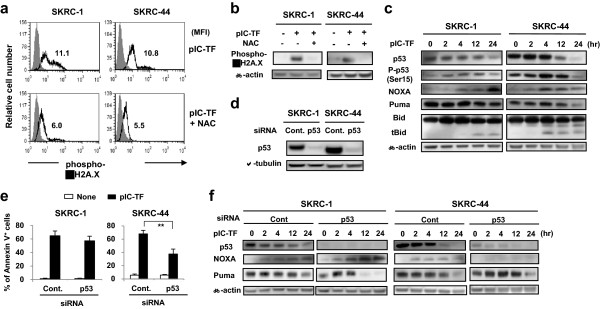
**DNA damage and p53 activation in poly(I:C)-transfected RCC cells.** SKRC-1 and SKRC-44 cells were transfected with poly(I:C) (SKRC-1, 1,000 ng/ml; SKRC-44, 500 ng/ml) and cultured for 24 h in the presence or absence of NAC (10 mM). **(a)** The levels of phosphorylated γH2AX (Ser 139) in untreated controls (gray) and poly(I:C)-transfected cells (bold line) were examined by flow cytometry. Numbers represent the mean fluorescence intensities (MFI) of poly(I:C)-transfected cells. Similar results were obtained from three independent experiments. **(b)** The level of phosphorylated γH2AX (Ser 139) was examined by immunoblotting. NAC (10 mM) was added 6 h after poly(I:C) transfection. β-actin was used as a positive control. Similar results were obtained from two independent experiments. **(c)** Kinetic levels of total p53, phosphorylated p53 (Ser 15), NOXA, Puma, Bid, and tBid were examined by immunoblotting. β-actin was used as a control. Similar results were obtained from two independent experiments. **(d)** P53 protein expression was examined by immunoblotting 3 days after transfection with control siRNA or p53 siRNA. α-tubulin was used as a control. **(e)** RCC cells, which were pre-transfected with the indicated siRNAs 3 days prior, were transfected additionally with poly(I:C). The percentage of Annexin V^+^ cells were then determined by flow cytometry after 24 h. **p < 0.01. Similar results were obtained from two independent experiments. **(f)** Kinetic levels of total p53, NOXA and Puma were examined by immunoblotting. β-actin was used as a control. Similar results were obtained from two independent experiments. pIC-TF, poly(I:C) transfection.

ROS induce DNA damage, and phosphorylation of histone H2A.X (γH2A.X) (Ser 139) is an indicator of DNA double-strand breaks. Therefore, we next determined whether DNA damage was induced in poly(I:C)-transfected RCC cells in a ROS-dependent manner. Figure 3a shows that poly(I:C) transfection increased phosphorylation of γH2A.X (Ser 139) in both cell lines, and that adding of NAC alleviated its expression. Similar results were observed in immunoblotting (Figure 3b). These results indicate that ROS are responsible for DNA damage in poly(I:C)-transfected RCC cells.

DNA damage induces p53 activation, which can ultimately lead to apoptosis [12, 20]. Therefore, we examined the levels of total and phosphorylated p53, as well as NOXA and Puma (p53 target molecules) in poly(I:C)-transfected RCC cells. Although p53 protein levels decreased 12 or 24 h after poly(I:C) transfection, phosphorylated p53 increased transiently in both cell lines from 2 to 12 h after poly(I:C) transfection, but decreased thereafter (Figure 3c), likely due to degradation of total p53. Although NOXA expression increased shortly after p53 activation, Puma expression began to decrease 12 h after poly(I:C) transfection, exhibiting similar kinetics to those of total p53. Additionally, tBid expression began to increase 4 or 12 h after poly(I:C) transfection. These results indicate that poly(I:C) transfection initially triggered p53 activation in conjunction with NOXA, but degraded p53 thereafter. Furthermore, poly(I:C) transfection caused the conversion of Bid into tBid, leading to caspase-8 activation in poly(I:C)-transfected RCC cells.

RNA interference was then performed to examine the role(s) of p53 in poly(I:C) transfection-induced apoptosis. Transfection of p53 siRNA decreased p53 protein expression in both cell lines (Figure 3d). Additionally, p53 knockdown decreased apoptosis in SKRC-44 cells, but not in SKRC-1 cells (Figure 3e). Moreover, the effects of p53 knockdown on p53 target molecules were also examined (Figure 3f). Although p53 knockdown increased NOXA expression slightly in poly(I:C)-transfected SKRC-1 cells, its expression was abolished in poly(I:C)-transfected SKRC-44 cells. Alternatively, p53 knockdown appeared to promote a decrease in Puma expression in poly(I:C)-transfected SKRC-1 cells, whereas no apparent change in Puma expression was observed in poly(I:C)-transfected SKRC-44 cells. These results indicate that apoptosis and NOXA expression are dependent on p53 activation after poly(I:C) transfection in SKRC-44 cells, but not in SKRC-1 cells.

### Caspase-dependent apoptosis in RCC cells after poly(I:C) transfection

**Figure 4:**
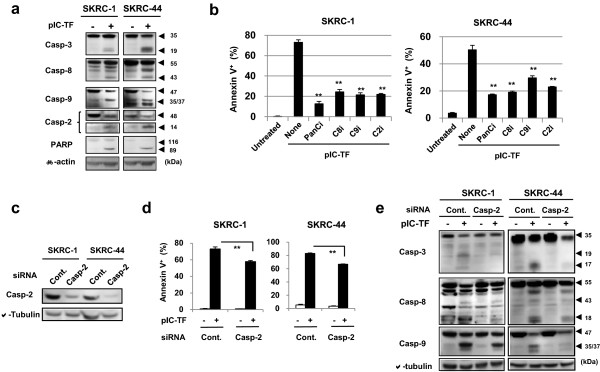
**Caspase-dependent apoptosis in poly(I:C)-transfected RCC cell lines. (a)** Two RCC lines were cultured or transfected with poly(I:C) (SKRC-1, 500 ng/ml; SKRC-44, 50 ng/ml). After 24 h, cleaved forms of a panel of caspases and PARP were evaluated by immunoblotting. β-actin was used as a control. Similar results were obtained from two independent experiments. **(b)** RCC lines were cultured or transfected with poly(I:C) (SKRC-1 and SKRC-44, 500 ng/ml) in the presence of the indicated caspase inhibitors (20 μM). The percentage of Annexin V^+^ cells was determined by flow cytometry. Results are presented as the means ± standard deviations of three wells. **p < 0.01 compared with poly(I:C) transfection alone. Similar results were obtained from two independent experiments. PanCi, pan-caspase inhibitor; C8i, caspase-8 inhibitor; C9i, caspase-9 inhibitor; C2i, caspase-2 inhibitor. **(c)** Caspase-2 protein expression was examined by immunoblotting 3 days after transfection with control siRNA or caspase-2 siRNA. α-tubulin was used as a control. **(d)** RCC cells, which were pre-transfected with the indicated siRNAs 3 days prior, were transfected additionally with poly(I:C) (SKRC-1, 1,000 ng/ml; SKRC-44, 500 ng/ml). The percentage of Annexin V^+^ cells was determined by flow cytometry after 24 h. Results are presented as the means ± standard deviations of three wells. **p < 0.01. Similar results were obtained from two independent experiments. **(e)** After poly(I:C) transfection (SKRC-1, 1,000 ng/ml; SKRC-44, 500 ng/ml), the expression of caspases was evaluated by immunoblotting. β-tubulin was used as a control. Similar results were obtained from two independent experiments. pIC-TF, poly(I:C) transfection.

Because caspases play a central role in apoptosis, we examined activation of caspases. Poly(I:C) transfection induced cleaved forms of caspase-3, -8, -9, -2, and poly (ADP-ribose) polymerase (PARP), a caspase substrate, in these cell lines (Figure 4a). We included caspase-2 because this caspase has been suggested to be involved in ROS-mediated apoptosis [15]. As shown in Figure 4b, adding of z-VAD-fmk, a pan-caspase inhibitor, apparently inhibited the increase in the percentages of Annexin V^+^ cells in poly(I:C)-transfected RCC cells. Although the inhibition levels varied, inhibitors of caspase-8, -9, or -2 also inhibited apoptosis in poly(I:C)-transfected RCC cells. To examine the influence of caspase-2 on poly(I:C) transfection-induced apoptosis and activation of other caspases, caspase-2 was knocked down selectively using caspase-2 siRNA (Figure 4c), which consequently decreased apoptosis (Figure 4d, Additional file 1: Figure S1), as well as caspase-3, -8, and -9 activation in poly(I:C)-transfected RCC cells (Figure 4e). These results indicate that caspase-8 and -9, as well as caspase-2, are involved in apoptosis in poly(I:C)-transfected RCC cells, and that caspase-2 influences the activation of capase-8 and -9.

### IFN-β-mediated growth arrest of poly(I:C)-transfected RCC cells

**Figure 5:**
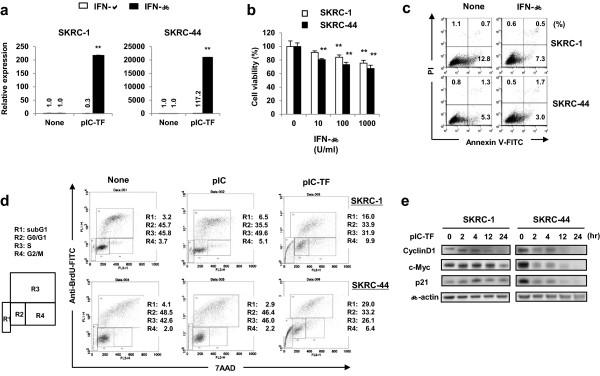
**Effects of IFN-β on RCC growth. (a)** Twenty-four hours after poly(I:C) transfection, cells were harvested and real-time PCR was performed to examine IFN-α and IFN-β mRNA expression. Relative mRNA levels are shown relative to those of β-actin. **p < 0.01. **(b)** RCC lines were co-cultured with human IFN-β, and cell viability was determined using the WST-8 assay after 24 h. **p < 0.01. **(c)** Similarly, after culturing with human IFN-β (1,000 U/ml), an apoptosis assay was performed using Annexin V and PI staining. Numbers represent the percentages of each subset. Similar results were obtained from three independent experiments. **(d)** Both cell lines were cultured or transfected with poly(I:C) (SKRC-1, 100 ng/ml; SKRC-44, 50 ng/ml) for 48 h. Cells were then cultured with BrdU (10 μM) during the last 90 min for SKRC-1 and 6 h for SKRC-44. After staining with FITC-conjugated anti-BrdU and 7-AAD, cells were analyzed by flow cytometry. Numbers represent the percentages for each cell cycle phase. Similar results were obtained from two independent experiments. **(e)** Levels of cyclinD1, c-Myc and p21 were examined by immunoblotting kinetically after poly(I:C) transfection. β-actin was used as a control. Similar results were obtained from two independent experiments. pIC-TF, poly(I:C) transfection.

We next tested the possibility that poly(I:C) transfection triggers IFN-β, thereby causing growth arrest, since poly(I:C) induces IFN-β production [22] and IFN-β has the potential to induce growth arrest in RCC cells [23]. Real-time PCR revealed that poly(I:C) transfection increased IFN-β mRNA expression strongly in both cell lines (Figure 5a). Treatment with recombinant IFN-β decreased viability in both cell lines, but no apparent apoptosis was observed (Figure 5b, c). We next examined the proliferation capacity of poly(I:C)-transfected RCC cells. As shown in Figure 5d, the proportion of bromodeoxyuridine (BrdU)^+^ S-phase cells was decreased in poly(I:C)-transfected RCC cells but increased in the apoptotic sub-G1 fraction. We also examined the effect of recombinant IFN-β on the cell cycle and determined that IFN-β similarly decreased S-phase cells in both cell lines (Addditional file 2: Figure S2). The expression levels of cell cycle-related molecules were examined subsequently, which revealed that poly(I:C) transfection decreased protein expression of cyclinD1 and c-Myc in both cell lines (Figure 5e). Although p21 expression transiently increased 4 h after poly(I:C) transfection in SKRC-1 cells, its expression decreased rapidly in SKRC-44 cells after poly(I:C) transfection. Taken together, these data suggest that IFN-β mediates cell growth arrest, decreases expression of cyclinD1 and c-Myc, and that both molecules play a role in decreasing the cell viability of poly(I:C)-transfected RCC cells.

### Innate adjuvant receptors and the 2-5A system on the effects of poly(I:C) transfection in RCC cells

**Figure 6:**
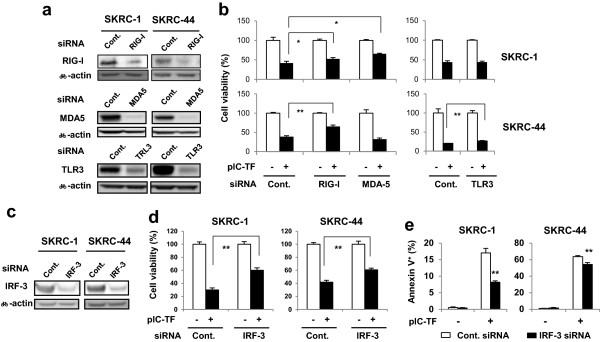
**Participation of innate adjuvant receptors in antitumor effects of poly(I:C) transfected RCC cells. (a)** RCC cells were transfected with RIG-1, MDA5, TLR3, or control siRNA, and selective knockdown was confirmed by immunoblot assay after 72 h. β-Actin was used as the control. **(b)** RCC cells, which were pre-transfected with the indicated siRNA 72 h before, were additionally transfected with poly(I:C) (125 ng/ml for SKRC-1 and 62.5 ng/ml for SKRC-44). Cell viability was determined by the WST-8 assay after 24 h. Similar results were obtained from two independent experiments. **(c)** The RCC cells were transfected with either IRF-3 or control siRNA, or selective knockdown was confirmed after 72 h by immunoblot assay. β-Actin was used as the control. **(d)** RCC cells, which were pre-transfected with the indicated siRNA 72 h before, were additionally transfected with poly(I:C) (250 ng/ml for SKRC-1 and 31.3 ng/ml for SKRC-44). Cell viability was determined by the WST-8 assay after 24 h. Similar results were obtained from two independent experiments. **(e)** RCC cells, which were pre-transfected with the indicated siRNA 72 h before, were additionally transfected with poly(I:C) (1000 ng/ml for SKRC-1 and 500 ng/ml for SKRC-44). The percentages of apoptotic cells were determined by flow cytometry after 24 h. Similar results were obtained from two independent experiments. pIC-TF, poly(I:C) transfection. *P < 0.05, **P < 0.01.

Transfection of poly(I:C) into human melanoma cells can induce cell death via the cytoplasmic poly(I:C) receptor MDA5 [9, 10]. We have also reported that poly(I:C) transfection exhibits antitumor effects in human breast cancer cells [11], but selective knockdown of MDA5 only partially recovers cell viability, suggesting that other systems are involved. Therefore, we next determined whether other innate adjuvant receptors and mechanisms might participate in the effects in poly(I:C)-transfected RCC cells. Selective knockdown of three innate adjuvant receptors, retinoic acid-inducible gen (RIG)-I, MDA5, and TLR3, was confirmed by immunoblotting (Figure 6a). Selective knockdown of RIG-I or MDA5 slightly but significantly restored viability of poly(I:C)-transfected SKRC-1 cells, and selective knockdown of RIG-I restored viability of poly(I:C)-transfected SKRC-44 cells (Figure 6b). Additionally, selective knockdown of TLR3 slightly restored viability of poly(I:C)-transfected SKRC-44 cells. In addition, we examined the effect of selective knockdown of interferon regulatory transcription factor (IRF)-3 on antitumor effects in poly(I:C)-transfected RCC cells, as IRF-3 is involved with the signaling pathways of these three adjuvant receptors. The result showed that selective knockdown of IRF-3 significantly restored cell viabilities (Figure 6c, d) and decreased Annexin V^+^ apoptotic cells (Figure 6e) in both cell lines. Recovery of apoptotic cells by the selective knockdown of IRF-3 after poly(I:C) transfection was more apparent in SKRC-1 cells than that in SKRC-44 cells.

**Figure 7:**
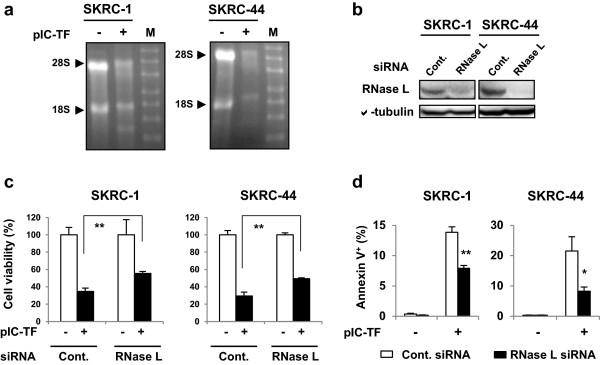
**Participation of the 2-5A system in the poly(I:C) transfection-induced antitumor effect. (a)** One day after poly(I:C) transfection (500 ng/ml), total RNA was extracted, and 4 μg total RNA from each sample was denatured and analyzed by electrophoresis using 1% agarose-formaldehyde gels, ethidium bromide, and UV light. Similar results were obtained from two independent experiments. M, marker. **(b)** RCC cells were then transfected with either RNaseL or control siRNA. After 72 h, selective knockdown was confirmed by immunoblotting. α-tubulin was used as a control. **(c)** RCC cells, which were pre-transfected with the indicated siRNAs 72 h prior, were transfected additionally with poly(I:C) (SKRC-1, 250 ng/ml; SKRC-44, 31.3 ng/ml). Cell viability was determined using a WST-8 assay after 24 h. Similar results were obtained from three independent experiments. **(d)** RCC cells, which were pre-transfected with the indicated siRNA 72 h prior, were transfected additionally with poly(I:C) (SKRC-1, 500 ng/ml; SKRC-44, 50 ng/ml). The proportion of apoptotic cells was determined by flow cytometry after 24 h. Similar results were obtained from three independent experiments. pIC-TF, poly(I:C) transfection. *p < 0.05, **p < 0.01.

Partial restoration of cell viability and apoptosis of poly(I:C)-transfected RCC cells by selective IRF-3 knockdown led us to search for an alternative mechanism. dsRNA activates 2-5OAS to produce 2-5A, thereby activating RNaseL [18]; this 2-5A system degrades viral RNA and induces apoptosis in virus-infected cells [19]. Therefore, we examined whether the 2-5A system participates in the effects observed after poly(I:C) transfection in RCC cells. As shown in Figure 7a, rRNA was cleaved in poly(I:C)-transfected RCC cells, suggesting that RNaseL was activated in RCC cells following poly(I:C) transfection. Selective knockdown of RNaseL was confirmed by immunoblotting (Figure 7b). Knockdown of RNaseL significantly restored cell viability and decreased Annexin V^+^ apoptotic cells in both cell lines (Figure 7c, d). Taken together, these results suggest that both innate adjuvant receptors and the 2-5A system participate in the poly(I:C) transfection-induced effects in RCC cell lines.

## Discussion

In this study, we revealed that poly(I:C) transfection induced apoptosis in human RCC cells. Caspases can be divided into two groups: initiator caspases, including caspase-8 and -9, and effector caspases, including caspase-3 and -7 [24]. Caspase-8 and -9 are mainly responsible for extrinsic and intrinsic caspase-dependent apoptosis, respectively [25]. In contrast, caspase-2 is unique in that it has features of both initiator and effector caspases [26], but the roles of caspase-2 in apoptosis remain largely unknown. In this study, we showed that adding a caspase-2 inhibitor diminished apoptosis in poly(I:C)-transfected RCC cells, and that selective knockdown of caspase-2 inhibited both apoptosis and activation of caspase-3, -8, and -9 in poly(I:C)-transfected RCC cells. These results suggest that caspase-2 plays crucial roles in apoptosis of poly(I:C)-transfected RCC cells via an effect on caspase-8 and -9. Alternatively, we showed that caspase-8 was activated in poly(I:C)-transfected RCC cells. Similarly, a previous report showed that both mitochondrial and caspase-8-dependent pathways are implicated in poly(I:C)-induced apoptosis in melanoma cells [27]. We showed that poly(I:C) transfection induced cleavage of Bid to its active form tBid (Figure 3c). Bid activation occurs through cleavage by caspase-8, which generates the truncated form, tBid [13]. As activation of caspase-8 is generally triggered by extrinsic death signals, a question remains as to how caspase-8 was activated in poly(I:C)-transfected RCC cells. Note that activation of caspase-2 has been reported to activate caspase-8, and sequential activation of caspase-2 and -8 is essential for saikosaponin a-induced apoptosis in human colon cancer cells [28, 29]. These findings suggest that activation of caspase-8 in poly(I:C)-transfected RCC cells resulted from activation of caspase-2, and that conversion of Bid to tBid via activation of caspase-2 or -8 contributed to stimulate mitochondria-mediated activation of caspase-9. Caspase-2 seemed to form a signaling link between the extrinsic apoptotic and intrinsic mitochondrial-based apoptotic pathways.

As described above, the extrinsic and intrinsic pathways converge at the mitochondrial level and trigger mitochondrial membrane permeabilization [21, 30]. Intrinsic apoptosis is mediated through a decrease in the ΔΨm, thereby resulting in the release of cytochrome *c* and activation of caspase-9. In this study, we tested the possibility that ROS were involved in this process because ROS are recognized as a central mediator in deciding cell fate [31]. Mitochondrial functions depend on the maintenance of ΔΨm, and loss of this potential leads to apoptosis [32]. In addition, mitochondrial production of ROS also appears to play a role in cell death [33]. In this study, we demonstrated that ROS increased in poly(I:C)-transfected RCC cells, and that NAC, a ROS scavenger, inhibited apoptosis in these cells. In addition, NAC restored the decreased ΔΨm, and apoptosis and the level of the ΔΨm were conversely correlated in poly(I:C)-transfected RCC cells (Figure 2d). Together, these findings indicate that poly(I:C) transfection induces ROS first and subsequently decreases the ΔΨm level, resulting in activation of caspase-9 and apoptosis.

Poly(I:C) transfection increased γH2A.X phosphorylation (Ser 139) in RCC cells (Figure 3a, b). Notably, inhibition of ROS with NAC inhibited its phosphorylation in poly(I:C)-transfected RCC cells, suggesting that poly(I:C) transfection induces ROS and subsequently leads to DNA damage, which induces apoptosis [34, 35]. In the study described herein, we showed that poly(I:C) transfection induced time-dependent increases in NOXA just after p53 activation (Figure 3c). Poly(I:C) treatment was reported previously to induce an interaction between NOXA and Bax, leading to mitochondrial apoptosis [36]. Puma is a pro-apoptotic protein that facilitates apoptosis via a wide variety of stimuli in p53-dependent and -independent manners [37]. In this study, poly(I:C) transfection slightly decreased Puma in the RCC lines (Figure 3c).

The cytoplasmic delivery of poly(I:C) induced ROS production in RCC cells (Figure 2a). Intriguingly, some reports suggest that DNA damage induces ROS production [15, 38]. Both DNA damage and ROS production may mutually affect this process, leading to augmentation of apoptosis. Importantly, ROS activate caspase-2, and DNA damage also induces cleavage of caspase-2 [39]. Caspase-2 is activated in response to DNA damage and provides an important link between DNA damage and engagement of the apoptotic pathway [15, 38]. Additionally, ROS trigger caspase-2 activation and induce apoptosis in a human leukemic T cell line [40]. Based on these data, ROS trigger DNA damage, thereby leading to activation of caspase-2. DNA damage also induces p53 activation, resulting in mitochondrial-mediated apoptosis.

IFN-α has been clinically applied to treat patients with RCC [41]. IFN-α shows biological effects similar to those of IFN-β because they share receptors. Poly(I:C) induces IFN-β production [22], and IFN-β mRNA expression increased in poly(I:C)-transfected RCC cells (Figure 5a). Therefore, we determined whether IFN-β showed an antitumor effect in RCC cells. Although no apoptosis was observed, an *in vitro* culture with IFN-β decreased the number of RCC cells (Figure 5b, c), suggesting that IFN-β shows an antitumor effect via cell-growth arrest, but not via apoptosis in RCC cells. Note that NOXA is a type-I IFN-response gene [36]. While both NOXA and Puma are p53-targeted molecules, NOXA expression increased following poly(I:C) transfection shortly after p53 activation, whereas Puma expression decreased, accompanying the decreased expression of total p53 (Figure 3c). Interestingly, p53 knockdown inhibited NOXA induction after poly(I:C) transfection in SKRC-44 cells, but not in SKRC-1 cells (Figure 3f). These results suggest that NOXA induction in SKRC-44 cells after poly(I:C) transfection is highly p53-dependent, but SKRC-1 cells are dependent on not p53 but the IFN-β response. Alternatively, induction of cell growth arrest occurs in response to various stressors including DNA damage [42]. This in turn allows for p53 nuclear translocation and activation of transcriptional targets such as p21^Waf1/Cip1^, a cyclin-dependent kinase inhibitor, to regulate cell cycle control and apoptosis [43]. Our results demonstrate that p21 expression increases transiently in poly(I:C)-transfected SKRC-1 cells, but decreases rapidly in poly(I:C) transfected SKRC-44 cells. G1 arrest was not obvious in the cell cycle assay, but poly(I:C) transfection decreased the proportion of RCC cells in the S phase (Figure 5d). In addition, cyclinD1 and c-Myc expression decreased after poly(I:C) transfection (Figure 5e). Moreover, recombinant IFN-β induced a growth arrest (Additional file 2: Figure S2). Taken together, poly(I:C) transfection appears to induce growth arrest via IFN-β as a result of suppressing the cell cycle accelerators cyclinD1 and c-Myc.

Transfection of a dsRNA poly(I:C) mimics viral infection. Cellular apoptosis after viral infection may represent an antiviral mechanism that eliminates infected cells and prevents viral spreading. In this study, we determined which innate adjuvant receptors, including TLR3, MDA5, and RIG-I, were responsible for the effects induced in poly(I:C)-transfected RCC cells. The result showed that selective knockdown of either RIG-I, MDA-5, or TLR3 slightly but significantly restored the decreased cell viabilities and decreased the percentages of apoptosis in poly(I:C)-transfected RCC cells. Additionally, selective knockdown of IRF-3 significantly recovered the viability of poly(I:C)-transfected RCC cells (Figure 6d, e). As IRF-3 is an adaptor molecule that is shared by all three innate adjuvant receptors [44], this result indicates that innate adjuvant receptors definitely participate in the effects of poly(I:C)-transfected RCC cells. Nevertheless, as mitigation of the effects by selective knockdown of IRF3 was partial, we searched for another pathway. Besides the innate adjuvant receptors, the 2-5A system is another system that induces apoptosis in virus-infected cells [17]. This system is related to mitochondrial apoptosis and IFN activates the 2–5 system [45]. In the present study, we showed that selective knockdown of RNaseL restored decreased cell viability and decreased the percentage of apoptotic cells of poly(I:C)-transfected RCC cells. This is the first report showing that the 2-5A system participates in apoptosis of poly(I:C)-transfected human cancer cells. Intriguingly, an RNase L mutation is associated with prostate cancer risk, suggesting that the 2-5A system works as a tumor suppressor [46].

## Conclusions

We revealed that poly(I:C) transfection induced ROS-mediated apoptosis and IFN-β-mediated cell-growth arrest in RCC cells. Besides caspase-8 and -9, caspase-2 was suggested to play important roles in the effects of poly(I:C)-transfected RCC cells. We further showed that not only the innate adjuvant receptors but also the 2-5A system takes part in this response. Our findings unveil new mechanisms underlying apoptosis and growth arrest of cancer cells after poly(I:C) transfection.

## Materials and methods

### Cell cultures and reagents

Two human renal cell carcinoma lines (SKRC-1 and SKRC-44) were kindly provided by Dr. K. Yoshikawa (Aichi Medical University) and maintained in RPMI-1640 medium (Sigma-Aldrich, St. Louis, MO, USA) supplemented with 10% fetal bovine serum (Sigma-Aldrich) and gentamicin (10 μg/ml) in a humidified 5% CO_2_ incubator at 37°C. Low-molecular-weight poly(I:C) was purchased from InvivoGen (San Diego, CA, USA). Recombinant human IFN-β was acquired from PeproTech (Rocky Hill, NJ, USA). The following inhibitors were added 2 h before poly(I:C) transfection at a dose of 20 μM to inhibit caspases: the pan-caspase inhibitor z-VAD-FMK (Enzo Life Sciences, Farmingdale, NY, USA), the caspase-8 inhibitor z-IETD-FMK (R&D Systems, Minneapolis, MN, USA), the caspase-9 inhibitor z-LEHD-FMK (R&D Systems), and the caspase-2 inhibitor z-VDVAD-FMK (R&D Systems). NAC was purchased from Sigma-Aldrich.

### *In vitro*poly(I:C) transfection

Poly(I:C) was transfected into cancer cells using X-tremeGENE siRNA transfection reagent (Roche Applied Science, Mannheim, Germany), according to the manufacturer’s instructions.

### Cell viability assay

Cell viability was analyzed using the WST-8 assay (Nacalai Tesque, Kyoto, Japan). At the end of the incubation, 10 μl of WST-8 solution was added to each well, and the plates were incubated for an additional 3 h. Absorbance of each well was measured at 560 nm using a microplate reader (Beckman Coulter, Brea, CA, USA).

### Microscopy

RCC cells, which were treated with or without poly(I:C), were cultured in 6-well plates for 24 h. Morphological changes in RCC cells were observed using an inverted microscope (Olympus Corp., Tokyo, Japan).

### Apoptosis assay

Apoptosis was examined using the Annexin V-FITC Apoptosis Detection Kit (BioVision, Mountain View, CA, USA) according to the manufacturer’s instructions. In brief, cells were stained with Annexin V-FITC and PI. Stained cells were analyzed using FACSCalibur (BD Biosciences, San Jose, CA, USA). Data were plotted with FCSExpress software (De Novo Software, Los Angeles, CA, USA).

### ROS measurement

Intracellular ROS were measured using carboxy-H_2_DCFDA (Molecular Probes, Carlsbad, CA, USA). Treated cells were cultured with carboxy-H_2_DCFDA (10 μM) for 30 min. Thereafter, the collected cells were analyzed by flow cytometry. NAC was added at a dose of 5 mM to scavenge ROS, 6 h after poly(I:C) transfection.

### Mitochondrial membrane potential (ΔΨm) assay

The cells were collected 24 h after poly(I:C) transfection in 6-well plates and incubated with MitoProbe™ DiOC_2_(3) (3, 3′-diethyloxacarbicyanine iodide, Molecular Probes) at a concentration of 8 nM for 30 min at 37°C. In some experiments, the treated cells were stained with Annexin V-APC (BD Pharmingen, San Jose, CA, USA), followed by DiOC_2_(3). These stained cells were examined by FACSCalibur.

### DNA damage measurements

Phosphorylation of the histone H2A family member H2A.X (γH2A.X) at Ser 139 was measured by flow cytometry. Briefly, cells were fixed and permeabilized using an IntraPrep kit (Beckman Coulter). The cells were incubated with Alexa Fluor 488-conjugated anti-phospho-γH2A.X (Ser 139) antibody (Cell Signaling Technology, Danvers, MA, USA) and analyzed by FACSCalibur.

### Immunoblotting

Cells were lysed in M-PER reagent (Pierce, Rockford, IL, USA) with protease inhibitor cocktail (Nacalai Tesque). Protein concentration was determined using the Coomassie Plus Protein Bradford Kit (Pierce). NuPAGE gels (4–12% or 12%; Life Technologies, Carlsbad, CA, USA) were used for protein separation, and the proteins were immobilized onto PVDF membranes (Life Technologies) using the iBlot transfer system (Life Technologies). The membranes were blocked for 30 min using TBST (Tris-base-saline with 0.1% Tween 20) containing BlockerBSA solution (1×) (Pierce). Incubations with primary antibodies were performed using TBST containing BlockerBSA (1×) overnight at 4°C. The following primary antibodies were used: anti-phospho-γH2A.X (Ser 139) (9718), anti-phospho-p53 (Ser 15) (9284), anti-Puma (4976), anti-Bid (2002), anti-caspase-3 (9668), anti-caspase-2 (2224), anti-PARP (9532), anti-cyclin D1 (2926 T), anti-p21^Waf1/Cip1^ (2947), anti-RIG-I (4200 T), anti-MDA5 (5321), and anti-TLR3 (6961) (all from Cell Signaling Technology); anti-p53 (sc-71817,), anti-NOXA (sc-30209), anti-IRF-3 (sc-9082), anti-RNase L (sc-23955), anti-α-tubulin (sc-5286) (all from Santa Cruz Biotechnology, Santa Cruz, CA, USA); anti-caspase-8 (M032-3; Medical and Biological Laboratories, Nagoya, Japan), anti-caspase-9 (M054-3; Medical and Biological Laboratories), anti-c-Myc (1472–1; Epitomics, Burlingame, CA, USA), and anti-β-actin (622102) (Biolegend, San Diego, CA, USA). After washing, the membranes were incubated for 30 min at room temperature with alkaline phosphatase-conjugated secondary antibody. Protein bands were visualized using CDP-star chemiluminescence and photographed with an LAS-4000 (FujiFilm, Tokyo, Japan).

### Cell cycle assay

Proliferation of cancer cells was evaluated using a BrdU/7-aminoactinomycin D (7AAD) Flow kit (BD Pharmingen) according to the manufacturer’s instructions. Stained samples were analyzed by FACSCalibur.

### Transfection of small interfering RNA (siRNA)

Transfection of siRNA was performed using Lipofectamine RNAiMAX (Life Technologies) according to the manufacturer’s instructions. The following siRNAs were used: TLR3 siRNA (6236; Cell Signaling Technology), control siRNA (6568; Cell Signaling Technology) caspase-2 siRNA (sc-29236), RIG-I siRNA (sc-61480), MDA5 siRNA (sc-6101), IRF-3 siRNA (sc-35710), RNase L siRNA (sc-45965) (all from Santa Cruz Biotechnology), and p53 siRNA (SI02655170, QIAGEN, Venlo, Netherlands). The cancer cells were used for experiments 3 days after siRNA transfection.

### Real-time PCR

Total RNAs were extracted using the PureLink RNA Mini Kit (Life Technologies) with DNase I treatment. cDNAs were synthesized using random primers and the SuperScript VILO cDNA Synthesis Kit (Life Technologies). The synthesized first-strand cDNA was amplified using Plantinum Tag DNA polymerase (Invitrogen) with EXPRESS SYBR GreenER qPCR SuperMixes (Invitrogen). Real-time PCR was carried out in duplicate using the ABI PRISM 7000 Sequence Detection System. Thermal cycling included an initial denaturation step of 2 min at 95°C, followed by 40 cycles of 95°C for 15 sec, and 60°C for 1 min. Relative mRNA levels as compared with β-actin were calculated. The following primers were used; IFN-α: forward, 5′-GTG AGG AAA TAC TTC CAA AGA ATC AC-3′; and reverse, 5′-TCT CAT GAT TTC TGC TCT GAC AA-3′; IFN-β: forward, 5′-AGC TGA AGC AGT TCA GAA G-3′; and reverse, 5′-AGT CTC ATT CCA GCC AGT GC-3′; β-actin: forward, 5′-GCG AGA AGA TGA CCC AGA TC-3′; and reverse, 5′-CCA GTG GTA CGG CCA GAG G-3′.

### RNA degradation assay

After RNA extraction, 4 μg total RNA from each reaction was denatured and analyzed by electrophoresis in 1% agarose-formaldehyde gels. Ethidium bromide and UV light revealed the location of the RNA bands within the gels.

### Statistical analyses

Data were evaluated statistically using the unpaired two-tailed Student’s *t-*test. A *P-*value < 0.05 was considered significant.

## Electronic supplementary material

Additional file 1: Figure S1: Effects of caspase-2 knockdown on apoptosis in poly(I:C) transfected RCC cells. Both cell lines, which were pre-transfected with control or caspase-2 siRNA 3 days prior, were transfected additionally with poly(I:C) (SKRC-1, 1,000 ng/ml; SKRC-44, 500 ng/ml). Cells were then stained with FITC-conjugated Annexin V and PI and analyzed by flow cytometry after 24 h. Numbers represent the percentages for each subset. pIC-TF, poly(I:C) transfection. (PPTX 330 KB)

Additional file 2: Figure S2: Effects of IFN-β on RCC growth. Both cell lines were cultured with or without IFN-β (1,000 U/ml) for 48 h. Cells were then cultured with BrdU (10 μM) during the last 90 min for SKRC-1 and 6 h for SKRC-44. After staining with FITC-conjugated anti-BrdU and 7-AAD, cells were analyzed by flow cytometry. Numbers represent the percentages for each cell cycle phase. (PPTX 188 KB)

## Abbreviations

BrdU: Bromodeoxyuridine
carboxy-H_2_DCFDA: 5-(and-6)-carboxy-2′, 7′-dichlorohydrofluorescein diacetate
dsRNA: Double-stranded RNA
IFN: Interferon
IRF: Interferon regulatory transcription factor
γH2A.X: Histone-H2A.X
MDA: Melanoma differentiation-associated gene
NAC: *N*-acetyl-l-cysteine
2-5OAS: 2′,5′ oligoadenylate synthetase
PARP: Poly (ADP-ribose) polymerase
PI: Propidium iodide
RCC: Renal cell carcinoma
RIG: Retinoic acid-inducible gene
RNase L: 2-5A-depemdent RNase
ROS: Reactive oxygen species
TLR: Toll-like receptor
ΔΨm: Mitochondrial membrane potential.
