# Supplementary material for: Transfection of poly(I:C) can induce reactive oxygen species-triggered apoptosis and interferon-β-mediated growth arrest in human renal cell carcinoma cells via innate adjuvant receptors and the 2-5A system
Source: Mol Cancer. 2014 Sep 17;13:217. doi: 10.1186/1476-4598-13-217 (PMC4174632; doi:10.1186/1476-4598-13-217)
Supplement: Supplementary file 1 — Additional file 1: Figure S1: Effects of caspase-2 knockdown on apoptosis in poly(I:C) transfected RCC cells. Both cell lines, which were pre-transfected with control or caspase-2 siRNA 3 days prior, were transfected additionally with poly(I:C) (SKRC-1, 1,000 ng/ml; SKRC-44, 500 ng/ml). Cells were then stained with FITC-conjugated Annexin V and PI and analyzed by flow cytometry after 24 h. Numbers represent the percentages for each subset. pIC-TF, poly(I:C) transfection. (PPTX 330 KB) [file 12943_2014_1417_MOESM1_ESM.pptx]

## Slide 1
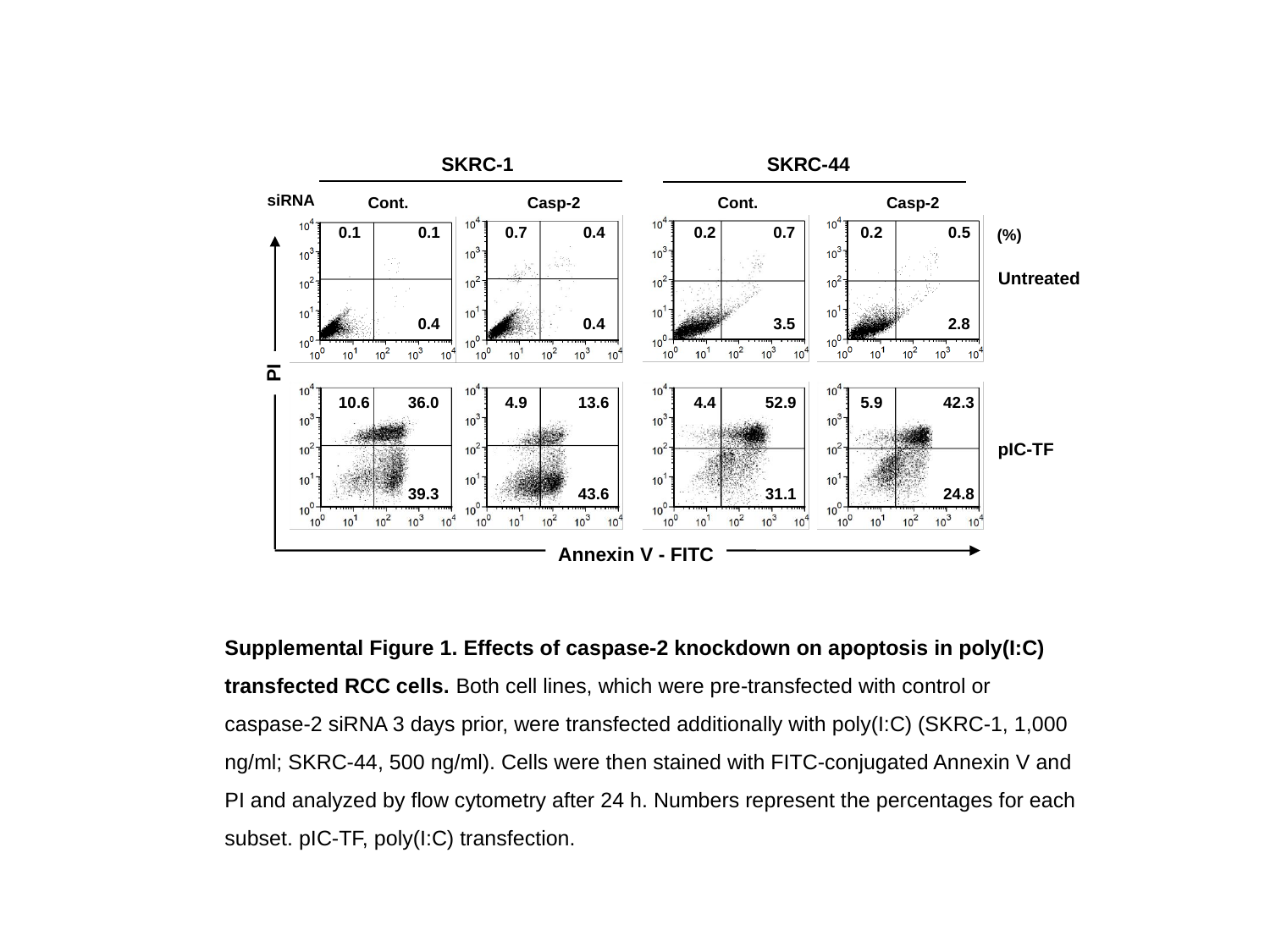

SKRC-1
SKRC-44
siRNA
Cont.
Casp-2
Cont.
Casp-2
0.2
0.7
0.2
0.5
0.1
0.1
0.7
0.4
(%)
Untreated
3.5
2.8
0.4
0.4
PI
4.4
52.9
5.9
42.3
10.6
36.0
4.9
13.6
pIC-TF
31.1
24.8
39.3
43.6
Annexin V - FITC
Supplemental Figure 1. Effects of caspase-2 knockdown on apoptosis in poly(I:C) transfected RCC cells. Both cell lines, which were pre-transfected with control or caspase-2 siRNA 3 days prior, were transfected additionally with poly(I:C) (SKRC-1, 1,000 ng/ml; SKRC-44, 500 ng/ml). Cells were then stained with FITC-conjugated Annexin V and PI and analyzed by flow cytometry after 24 h. Numbers represent the percentages for each subset. pIC-TF, poly(I:C) transfection.
