# Supplementary material for: Transfection of poly(I:C) can induce reactive oxygen species-triggered apoptosis and interferon-β-mediated growth arrest in human renal cell carcinoma cells via innate adjuvant receptors and the 2-5A system
Source: Mol Cancer. 2014 Sep 17;13:217. doi: 10.1186/1476-4598-13-217 (PMC4174632; doi:10.1186/1476-4598-13-217)
Supplement: Supplementary file 2 — Additional file 2: Figure S2: Effects of IFN-β on RCC growth. Both cell lines were cultured with or without IFN-β (1,000 U/ml) for 48 h. Cells were then cultured with BrdU (10 μM) during the last 90 min for SKRC-1 and 6 h for SKRC-44. After staining with FITC-conjugated anti-BrdU and 7-AAD, cells were analyzed by flow cytometry. Numbers represent the percentages for each cell cycle phase. (PPTX 188 KB) [file 12943_2014_1417_MOESM2_ESM.pptx]

## Slide 1
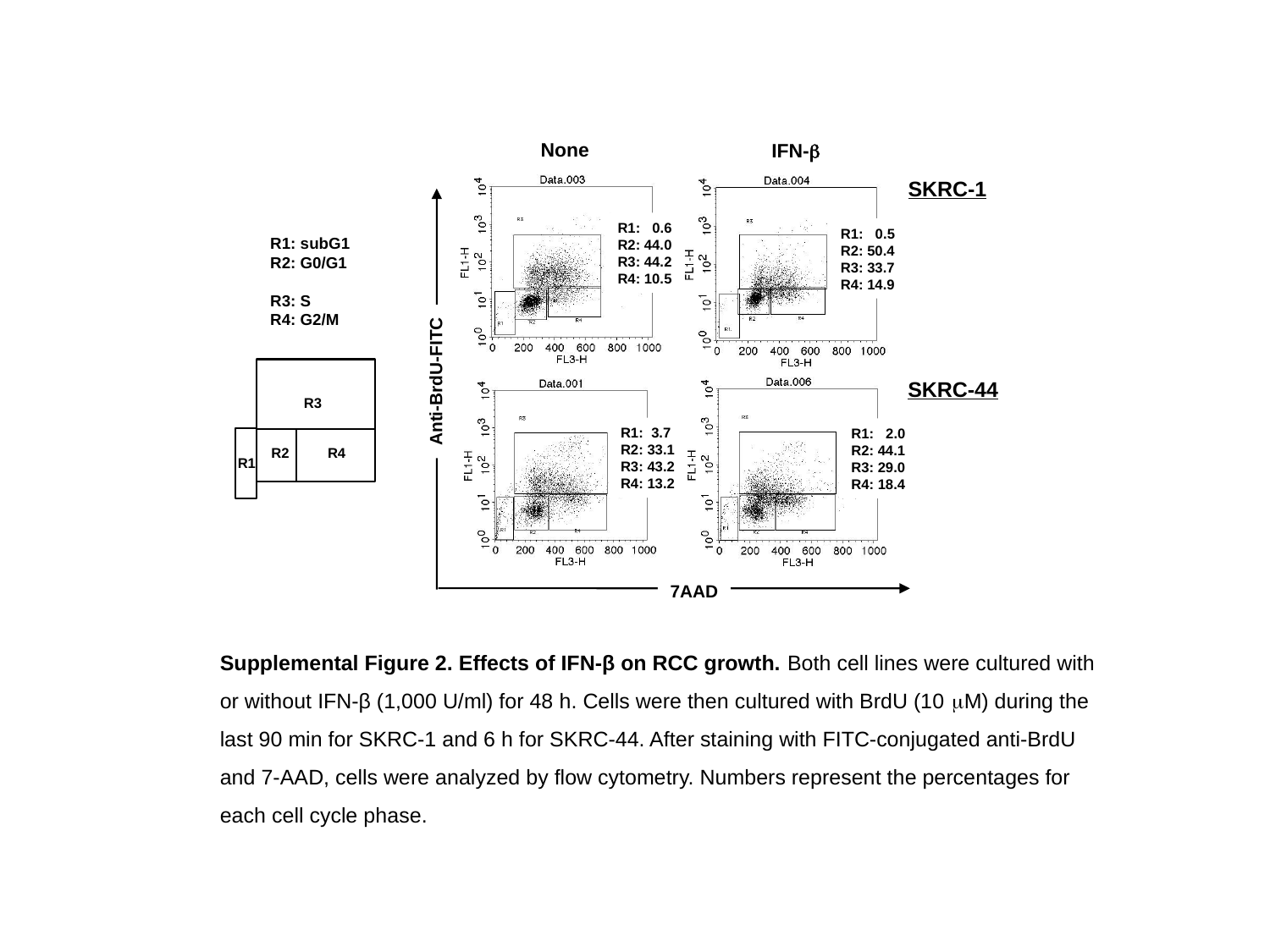

None
IFN-b
SKRC-1
R1: 0.6
R2: 44.0
R3: 44.2
R4: 10.5
R1: 0.5
R2: 50.4
R3: 33.7
R4: 14.9
R1: subG1
R2: G0/G1
R3: S
R4: G2/M
R3
R2
R4
R1
Anti-BrdU-FITC
SKRC-44
R1: 3.7
R2: 33.1
R3: 43.2
R4: 13.2
R1: 2.0
R2: 44.1
R3: 29.0
R4: 18.4
7AAD
Supplemental Figure 2. Effects of IFN-β on RCC growth. Both cell lines were cultured with or without IFN-β (1,000 U/ml) for 48 h. Cells were then cultured with BrdU (10 mM) during the last 90 min for SKRC-1 and 6 h for SKRC-44. After staining with FITC-conjugated anti-BrdU and 7-AAD, cells were analyzed by flow cytometry. Numbers represent the percentages for each cell cycle phase.
